# Transcriptional Regulation of *WTAP* Isoforms by NF-κB Signaling in Human Monocytes

**DOI:** 10.3390/ijms26199364

**Published:** 2025-09-25

**Authors:** Lucas W. Picavet, Hisham I. Abu-Tawil, Lyanne J. P. M. Sijbers, Jorg J. A. Calis, Nienke ter Haar, Alejandra Bodelón, Sebastiaan J. Vastert, Jorg van Loosdregt

**Affiliations:** 1 Center for Translational Immunology, University Medical Center Utrecht, 3584 EA Utrecht, The Netherlands; luuk.pica@gmail.com (L.W.P.); h.i.m.abutawil@umcutrecht.nl (H.I.A.-T.); a.bodelondefrutos@umcutrecht.nl (A.B.); b.vastert@umcutrecht.nl (S.J.V.); 2 Department of Laboratory and Blood Bank, King Faisal Medical City for Southern Regions, Ministry of Health, Abha 62523, Saudi Arabia; 3 Department of Pediatric Rheumatology and Immunology, University Medical Center Utrecht, 3584 EA Utrecht, The Netherlands

**Keywords:** WTAP, m6A, monocyte activation, NF-κB signaling, isoforms, LPS, RNA methylation, inflammation

## Abstract

N6-methyladenosine (m6A) is a dynamic RNA modification that critically modulates gene expression in immune responses. While m6A regulators such as WTAP are implicated in inflammatory and autoimmune diseases, the mechanisms governing their expression during innate immune activation remain unclear. Here, we demonstrate that *WTAP* expression in human CD14^+^ monocytes is upregulated upon lipopolysaccharide (LPS) stimulation and is associated with alternative promoter usage leading to distinct mRNA isoforms. Bioinformatic analysis and pharmacological inhibition reveal that the transcription factor RELA (NF-κB pathway) directly contributes to this promoter-specific induction. Functional analyses show that both *WTAP* isoforms encode identical proteins, indicating transcriptional, rather than post-transcriptional, regulation. These findings uncover a novel NF-κB-dependent mechanism regulating *WTAP* isoform expression in activated monocytes, providing insight into the epitranscriptomic modulation of inflammation and potential dysregulation in autoimmune disease.

## 1. Introduction

Post-transcriptional regulation through N6-methyladenosine (m6A) plays a crucial role in the regulation of gene expression in eukaryotic cells. m6A, the predominant RNA modification on mRNA, exerts multifaceted influence across various biological systems, encompassing metabolism, oncogenesis, and immunological responses. Orchestrated by the methyltransferase complex which includes METTL3, METTL14, and WTAP, m6A deposition is reversed by the demethylases FTO and ALKBH5. m6A and its regulatory constituents have been intricately implicated in inflammation and autoimmune disorders [1]. Upon microglial activation by lipopolysaccharide (LPS), METTL3 expression surged concomitantly with inflammatory cytokines, and its interaction with TRAF6 elicited NF-κB pathway activation in an m6A-dependent manner [2]. Similarly, LPS-stimulated macrophages exhibited heightened expression of the m6A reader YTHDF2, whose depletion augmented NF-κB and mitogen-activated protein kinase (MAPK) signaling alongside elevated expression of IL6, TNF, IL1β, and IL12 [3].

Previous studies have demonstrated that m6A methylation plays an essential role in monocyte and macrophage activation [4,5]. Specifically, METTL3 has been shown to be required for LPS-induced TNF production in macrophages, and elevated WTAP expression was found to enhance inflammation by increasing m6A modification in inflammatory disease models [4,5,6,7]. Moreover, recent studies have shown that m6A regulators such as METTL3 and METTL14 are upregulated in activated monocytes, where they promote pro-inflammatory gene expression via NF-κB signaling [8]. These findings support the role of m6A machinery, including WTAP, in modulating innate immune responses. Aberrant expression patterns of WTAP and other m6A-related proteins have been documented in autoimmune diseases such as rheumatoid arthritis (RA), multiple sclerosis, and systemic lupus erythematosus [9,10,11,12,13,14,15]. For example, reduced expression of m6A demethylases FTO and ALKBH5, coupled with diminished YTHDF2 levels in the blood of rheumatoid arthritis (RA)patients, have been identified as disease susceptibility factors [11]. However, the regulatory mechanisms governing the expression of m6A-associated proteins and WTAP during monocyte activation remain unknown.

In this study, we identified multiple *WTAP* mRNA isoforms within human CD14+ monocytes, linked to distinct promoters. Upon LPS-induced monocyte activation, transcription levels specifically increased for promoter-specific isoforms. Additionally, analysis unveiled the potential binding of the NF-κB subunit RELA to the WTAP promoter region and inhibition of NF-κB signaling attenuated the elevated WTAP expression seen in monocyte activation. Collectively, these findings highlight the role of NF-κB signaling and NF-κB in orchestrating the transcriptional regulation of select WTAP isoforms during monocyte activation.

## 2. Results

### 2.1. Alternative Promoter Utilization of WTAP in Human CD14^+^ Monocyte Activation

**Figure 1:**
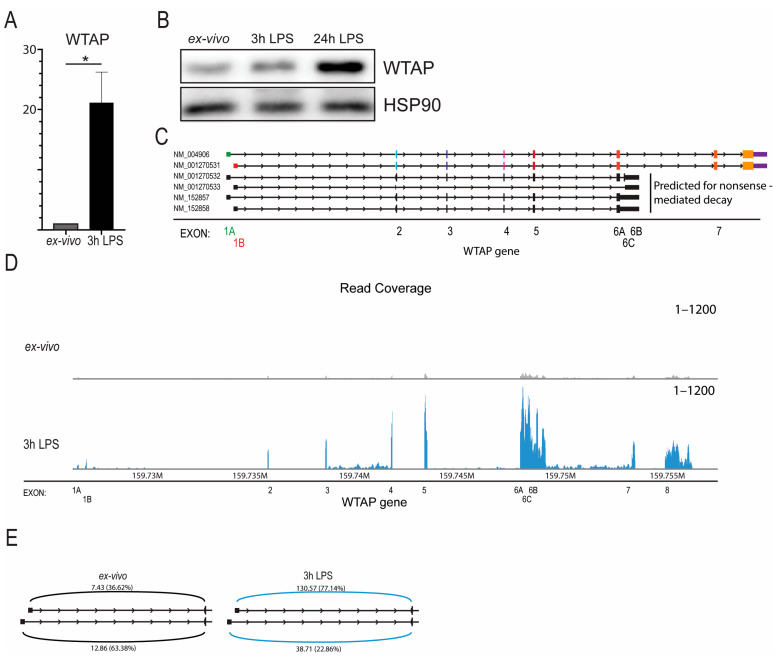
*WTAP* Expression increased in activated monocytes and is associated with alternative promoter usage. CD14^+^ Primary human monocytes activated with 100 ng/mL LPS for 3 h. (**A**) qPCR analysis of total *WTAP* mRNA expression (*n* = 3). (**B**) Western blot analysis of WTAP protein expression (*n* = 4). (**C**) *WTAP* mRNA isoforms as annotated by ENSEMBL. Exon 1A is shown in green and Exon 1B in red to distinguish the alternative isoforms. (**D**) Analysis of RNA-sequencing exon coverage of the *WTAP* gene (*n* = 7). (**E**) Sashimi plot displaying the average number of reads with coverage of *WTAP* exon 1A or exon 1B to exon 2 of monocytes ex vivo or with LPS stimulation derived from next-generation sequencing (*n* = 7). Statistical analysis by unpaired Student’s *t*-test; * *p* < 0.05.

To determine the role of WTAP in monocyte activation, human primary CD14^+^ monocytes were isolated from blood from healthy controls and activated with LPS for three hours. LPS stimulation resulted in increased expression of WTAP at the mRNA level (Figure 1A) and at protein level (Figure 1B). We further validated these results using RNA-sequencing data from human monocytes that we stimulated for three hours with LPS. Again we observed a strong induction of *WTAP* expression after LPS stimulation, while other components of the m6A writer complex *METTL3* and *METTL14* did not change their expression (Supplementary Figure S1). Subsequently, we aimed to understand the underlying mechanism responsible for the transcriptional initiation of WTAP. An examination of annotated *WTAP* transcripts in ENSEMBL revealed six distinct isoforms, originating from two alternative transcriptional start sites: exon 1A and exon 1B. Among these, only one isoform from each start site encodes the full-length WTAP protein (Figure 1C). To assess the expression of these exact *WTAP* mRNA isoforms, we analyzed next-generation RNA-sequencing dataset of human CD14^+^ monocytes subjected to LPS activation and utilized isoform-unique reads for isoform quantification. We identified read coverage for all annotated *WTAP* exons including expression of both exon 1A and exon 1B (Figure 1D). Interestingly, we identified a shift in isoform usage of *WTAP* where exon 1B was increasingly used after LPS stimulation (Figure 1E).

**Figure 2:**
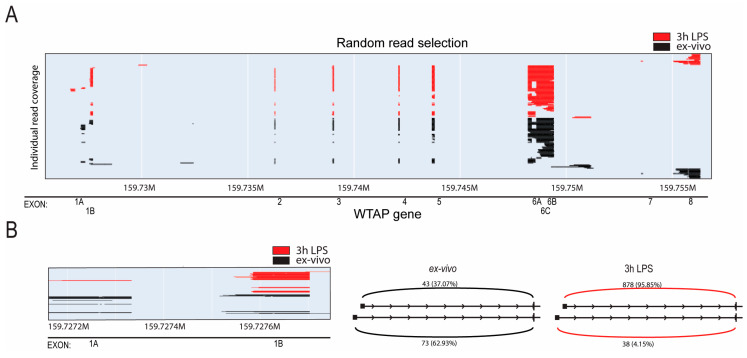
Isoform-specific regulation of *WTAP* revealed by Nanopore sequencing and qPCR validation. (**A**) *WTAP* exon coverage of first 100 long-read nanopore sequencing reads of ex vivo and LPS stimulated monocytes (*n* = 3). (**B**) Sashimi plot displaying the average number of reads with coverage of *WTAP* exon 1A or exon 1B to exon 2 of monocytes ex vivo or with LPS stimulation derived from nanopore sequencing (*n* = 3).

To further validate this observation, we employed direct long-read RNA sequencing (Oxford Nanopore Technologies) to be able to sequence whole transcripts. Again, we identified increased expression of *WTAP* isoforms containing exon 1B after LPS stimulation, evident through reads commencing further downstream in the gene (Figure 2A,B, Supplementary Figure S1A). Additionally, quantification of reads originating from either exon 1A or exon 1B in both sequencing datasets demonstrated an increased prevalence of exon 1B-containing isoforms post LPS stimulation (Supplementary Figure S1B). This observation was further validated through quantitative PCR utilizing primers specific to each exon (Supplementary Figure S1C). Notably, all three isoforms initiating from exon 1B exhibited similar upregulation upon LPS stimulation, with no evidence of differential usage of 3′ ends. This suggests a coordinated activation of the exon 1B promoter rather than selective expression of individual isoforms. In conclusion, our findings collectively indicate that *WTAP* expression in monocytes is increased after activation, which correlates to a shift in expressed *WTAP* originating from distinct promoters.

### 2.2. Transcription Factor RelA Promotes Transcriptional Initiation of Specific WTAP Isoforms in Monocyte Activation

**Figure 3:**
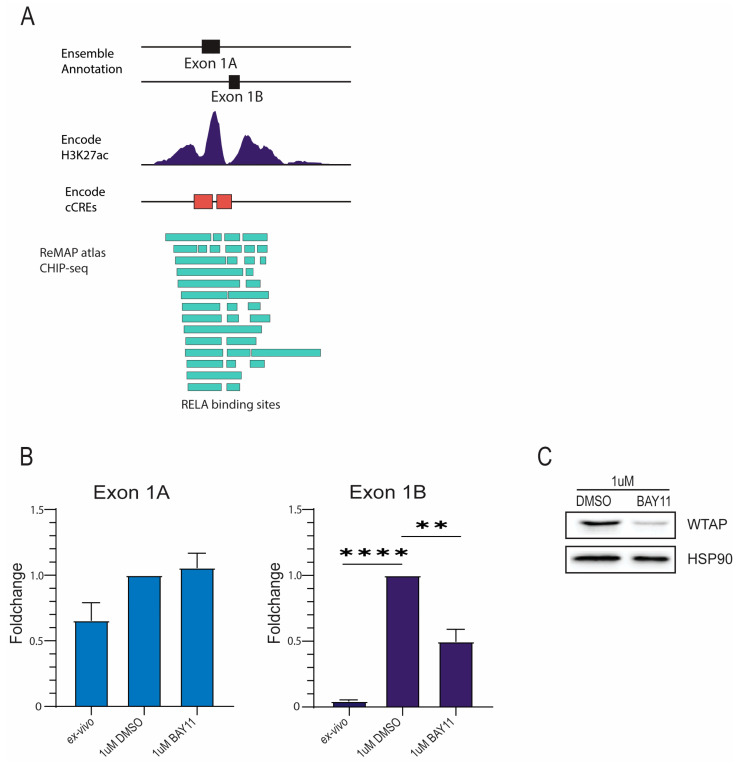
Increased WTAP expression in monocyte activation is regulated through NF-κB signaling. (**A**) Adjusted image from the UCSC gene browser displaying *WTAP* exon 1A and exon 1B region together with available H3K27ac data (ENCODE) annotated promoter regions (ENCODE) and annotated CHIP binding sites of RelA (ReMAP atlas). (**B**) qPCR analysis of *WTAP* exon 1A and exon 1B expression in activated monocytes treated with 1 µM BAY11 or dimethyl sulfoxide (DMSO) controls (*n* = 3). (**C**) Western blot analysis of WTAP protein in activated monocytes treated with 1 µM BAY11 or DMSO controls (*n* = 4). Statistical significance was assessed using one-way ANOVA (; ** *p* < 0.01; **** *p* < 0.0001).

Considering that LPS stimulation activates NF-κB signaling in monocytes, and that WTAP was recently identified as a transcriptional target of the NF-κB subunit p65 (RELA) [16], we investigated whether this pathway contributes to the transcriptional regulation of specific WTAP isoforms following monocyte activation. Subsequently, we aimed to understand the transcriptional regulation of the specific *WTAP* isoforms. We focused on potential transcription factor binding sites within the promoter region of *WTAP* isoform containing exon 1B (*WTAP1B*) for which transcription was increased after monocyte activation. The JASPAR Transcription Factor Database was used to identify transcription factors binding the *WTAP1B* promoter region [17]. This database contains a comprehensive and updated overview of transcription factor binding sites with additional transcription factor profiles. Potential binding sites for RELA, a subunit of the NF-κB complex, were identified in proximity to the promoter region indicating the involvement of RELA as the transcriptional activator for the initiation of *WTAP* transcription (Figure 3A). To test this, an inhibitor of NF-κB signaling (BAY 11-7082) was used [18]. BAY 11-7082 inhibits the translocation of the NF-κB subunit p65 thereby preventing transcriptional activity. Pretreatment of monocytes with BAY 11-7082 reduced the LPS-induced expression of *WTAP1B* transcripts (Figure 3B), resulting in reduced WTAP protein expression (Figure 3C). Taken together, these data suggest that NF-κB can promote WTAP transcription by binding to the promoter of the *WTAP1B* isoform after monocyte activation. In line with effective NF-κB inhibition, BAY 11-7082 treatment also reduced TNF expression in LPS-activated monocytes across multiple donors (Supplementary Figure S2), thereby providing validation that the inhibitor was functionally active in our system.

**Figure 4:**
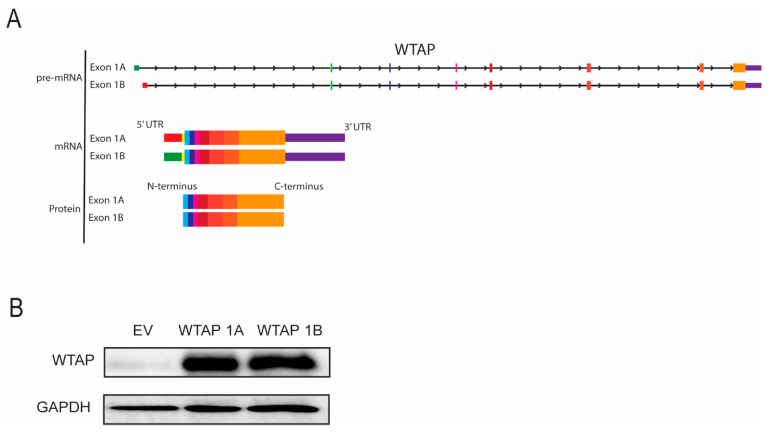
Both exon 1A and exon 1B containing *WTAP* mRNA isoforms lead to the same WTAP protein. (**A**) Schematic representation of exon 1A or exon 1B containing *WTAP* isoforms on pre-mRNA, mature mRNA and protein level. (**B**) Western blot analysis of WTAP protein expression in HEK293T cells transfected with empty vector (EV), WTAP 1A, or WTAP 1B isoforms. GAPDH was used as a loading control (*n* = 6).

To evaluate whether *WTAP* isoforms originating from exon 1A or exon 1B result in different protein products, structural composition was analyzed. As shown in the schematic (Figure 4A), both transcripts share an identical coding sequence (with alternative 5′UTR’s), indicative that they would produce an identical protein. To validate this, cells were transfected with constructs encoding full-length WTAP isoforms containing either exon 1A or exon 1B, including their 5′ and 3′ UTRs, under the control of the same promoter. Protein expression was assessed by Western blot. Indeed, both isoforms showed similar WTAP protein expression (Figure 4B). These results confirm that, although transcription is initiated from distinct promoters, both *WTAP* mRNA isoforms produce the same protein products. Thus, both isoforms encode identical WTAP protein products, indicating that functional differences between isoforms are not expected.

## 3. Discussion

WTAP plays a pivotal role as a component of the m6A methylase complex, crucial for orchestrating m6A deposition, with established functions in innate immunity encompassing the facilitation of immune responses and modulation of inflammatory processes. However, our comprehension of the regulatory mechanisms governing WTAP transcription remains limited. In this study, we have explored the regulatory influence of NF-κB signaling on the enhancement of WTAP expression during monocyte activation. Using long-read sequencing, we identified distinct *WTAP* isoforms transcribed from separate promoters. Upon monocyte activation, only the isoform originating from exon 1B (*WTAP1B*) was upregulated, whereas the exon 1A isoform remained unchanged. Furthermore, we have identified NF-κB signaling as a positive regulator of isoform-specific WTAP transcription. This initiation process is likely mediated by the binding of RELA to the promoter region of WTAP. Notably, *WTAP* 1A and 1B share an identical coding sequence and thus produce the same protein (Figure 4); therefore, our findings reflect transcriptional regulation rather than isoform-specific functional divergence. Previous studies have shown that WTAP expression itself is essential for monocyte and macrophage activation and cytokine signaling [4,7,16]. Thus, our results highlight NF-κB–mediated regulation of WTAP as the central mechanism. This observation suggests that the isoform switch does not alter WTAP protein structure, but rather reflects a layer of transcriptional regulation, potentially fine-tuning WTAP expression in response to inflammatory cues. WTAP exhibits widespread expression in a diverse array of human tissues. Its expression is notably heightened in various forms of cancer, encompassing renal, pancreatic, breast, and colorectal cancer, among others [19]. Furthermore, elevated WTAP expression is observed in inflammation and autoimmune disease.

Our findings underscore the broader concept that isoform-specific regulation, even in the absence of protein-coding differences, may represent a fundamental strategy for fine-tuning gene expression in immune cells. Alternative promoter usage can alter 5′ untranslated regions (UTRs), which in turn influence mRNA stability, secondary structure, and translation efficiency. In the context of monocyte activation, the selective induction of *WTAP* isoforms from exon 1B may therefore provide a mechanism to rapidly adjust *WTAP* expression kinetics in response to inflammatory cues without requiring changes in protein sequence. This suggests that promoter choice could serve as an additional regulatory layer in innate immunity, complementing post-transcriptional and post-translational mechanisms. Future studies should address whether such isoform-specific transcriptional programs are broadly deployed across m6A regulators or other immune-related genes, thereby contributing to the temporal precision of inflammatory responses. The transcriptional activity of WTAP is driven by the acetylation of histone H3 lysine 27 (H3K27), mediated by the CREB-binding protein (CBP), thereby implicating histone modification in the regulation of WTAP’s transcriptional processes. Moreover, the regulation of *WTAP* expression involves the participation of non-coding RNAs, which have well-established roles in disease severity. For example, the interaction between piwi RNA PiRNA-30473 and the 3′ untranslated region (3′ UTR) of *WTAP* mRNA, an interaction that enhances RNA stability [20]. Similarly, long non-coding RNA (lncRNA) PCGEM1 competes with microRNA miR433-3P to upregulate WTAP expression in lung cancer [21]. Analogously, LINC00839 in hepatocellular carcinoma and lncSNHG10 in osteosarcoma heighten WTAP expression by competitively binding to miR-144-3p [22,23]. Lastly, in pancreatic cancer, the lncRNA DUXAP8 binds competitively to miR-448, leading to an upregulation of WTAP expression [24].

Furthermore, WTAP protein expression is regulated post-transcriptionally. For instance, heat shock protein 90 stabilizes WTAP protein by inhibiting the ubiquitin-proteasome pathway [25]. Wilms tumor 1-associated protein pseudogene 1 (WTAPP1) promotes WTAP translation by facilitating the recruitment of the EIF complex [26]. In the context of breast cancer, the phosphorylation of WTAP by extracellular signal-regulated kinase 1 (ERK1) and extracellular signal-regulated kinase 2 (ERK2) at serine 341 enhances WTAP protein stability [27]. Additionally, signaling via the target of rapamycin complex 1 (mTORC1) is known to elevate WTAP protein expression, thereby fostering eIF4-mediated translation of *WTAP* mRNA [28].

Our study has revealed a novel mechanism underlying the regulation of WTAP expression in monocytes, involving the binding of the transcription factor RELA to an isoform-specific WTAP promoter. This is in contrast with previous research which demonstrated the dampening of WTAP expression through NF-κB signaling in the specific context of Epstein–Barr virus-associated gastric carcinoma (EBVaGC) [29]. The reduction in NF-κB signaling, achieved through interventions such as BAY 11-7085 treatment or targeted siRNA-mediated knockdown of p65, resulted in a significant increase in WTAP expression within the EBVaGC cell line GT39 [29]. Given the multitude of regulatory mechanisms governing WTAP expression, it is plausible that the regulation of WTAP expression varies between different cells and is markedly influenced by extracellular cues and the internal state of the cells. Further research is necessary to fully understand the way in which NF-κB signaling influences WTAP expression.

The identification of NF-κB as a direct transcriptional driver of WTAP also has important implications for human disease. Both NF-κB signaling and aberrant m6A regulation have been implicated in autoimmune disorders, including rheumatoid arthritis and systemic lupus erythematosus, as well as in inflammation-associated cancers. Our data raise the possibility that NF-κB–dependent induction of WTAP may amplify pro-inflammatory gene expression programs via enhanced m6A deposition, thereby fueling pathogenic immune activation in chronic inflammation. Conversely, in the tumor microenvironment, this axis may contribute to cancer cell survival and adaptation by reinforcing epitranscriptomic control of oncogenic transcripts. Therapeutically, promoter-specific regulation of WTAP offers an attractive angle; rather than complete NF-κB inhibition, which carries significant toxicity, selective modulation of WTAP expression or promoter accessibility may represent a more refined strategy to mitigate pathological inflammation while preserving essential host defense functions.

Collectively, the identification of isoform-specific WTAP regulation during monocyte activation provides new insights into how m6A modifications may be involved in inflammatory and cancer-related processes. The engagement of TLR4/NF-κB signaling in enhancing WTAP within monocyte activation unveils a potential mechanism for augmenting heightened m6A modifications, previously established as essential for pro-inflammatory signaling and TNF expression. Our study improves our understanding of the transcriptional regulation of WTAP in inflammation and may possibly contribute to understanding the mechanisms that underlie auto-immune diseases.

## 4. Materials and Methods

### 4.1. Cell Culturing

Peripheral blood mononuclear cells (PBMCs) were isolated from peripheral blood or buffy coats of healthy donors provided by Sanquin Blood Supply Foundation (Amsterdam, The Netherlands), using density gradient centrifugation with Ficoll-Paque™ PLUS (GE Healthcare, Chicago, IL, USA). CD14^+^ monocytes were purified using CD14 MicroBeads (Miltenyi Biotec, Bergisch Gladbach, Germany) according to the manufacturer’s protocol. Monocytes were either directly lysed or stimulated with 100 ng/mL lipopolysaccharide (LPS; Sigma-Aldrich, St. Louis, MO, USA) in RPMI 1640 medium (Thermo Fisher Scientific, Waltham, MA, USA) supplemented with 10% fetal bovine serum (FBS; Thermo Fisher Scientific, Waltham, MA, USA), 5 mM L-glutamine (Thermo Fisher Scientific, Waltham, MA, USA), and 1% penicillin-streptomycin (Thermo Fisher Scientific, Waltham, MA, USA), and incubated for 3 h at 37 °C in 5% CO_2_ prior to RNA or protein analysis.

### 4.2. qPCR

Total RNA was isolated using the RNeasy Mini Kit (Qiagen, Hilden, Germany). Complementary DNA (cDNA) was synthesized using the iScript™ cDNA Synthesis Kit (Bio-Rad, Hercules, CA, USA), and real-time quantitative PCR (qPCR) was performed using the SYBR™ Select Master Mix (Thermo Fisher Scientific, Waltham, MA, USA) according to the manufacturer’s protocols. The following primer sets were used to detect specific *WTAP* isoforms and the housekeeping gene *B2M*: *WTAP* Exon1A (fw: AAAGGACGGGGAGTGTTACC, rev: TGGGAAGAGGTTC TTCGTTGG), *WTAP* Exon1B (fw: AAATAAAGGGGAGCGCAGGG, rev: GGGAAGAGGTTCTTCGTTGG), *WTAP* Exon6B (fw: GGATTTCACAGGGAGGGCAA, rev: ACCCCGCACTGAGTTGATTT), *WTAP* Exon6C (fw: ATGGTAGACCCAGCGATCAAC, rev: GTGTCTACATGTTCTAGAAGGGGA), *WTAP* Exon8 (rev: CGAGTACTTGGCTGTCCCAC), *WTAP* all (fw: ATGGTAGACCCAGCGATCAAC, rev: TGCGACTAGCAACCAAGGAA or GTTGATCGCTGGGTCTACCA), and *B2M* (fw: TGCTGTCTCCATGTTTGATGTATCT, rev: TCTCTGCTCCCCACCTCTAAGT). Gene expression was normalized to *B2M* using the ΔCt method. All reactions were run in technical duplicates or triplicates.

### 4.3. Western Blot

Cells were lysed in Laemmli buffer (0.12 M Tris-HCl, pH 6.8, 4% SDS, 20% glycerol, 0.05 µg/µL bromophenol blue, 35 mM β-mercaptoethanol) and protein levels were normalized using the BCA protein assay kit (Thermo Fisher Scientific, Waltham, MA, USA). SDS-PAGE separation was performed with a 10% gel and transferred to a polyvinylidene difluoride (PVDF) membrane (Merck, Darmstadt, Germany). Membranes were incubated overnight at 4 °C with primary antibodies: rabbit anti-human WTAP (Abcam, Cambridge, UK, ab195380), rabbit anti-human HSP90 (Abcam, Cambridge, UK, ab13495), and mouse anti-GAPDH (Life Technologies, Carlsbad, CA, USA, MA5-15738). After washing, membranes were incubated with HRP-conjugated anti-rabbit or anti-mouse secondary antibodies (Agilent, Santa Clara, CA, USA, P026002-2), and signals were detected using chemiluminescence (Thermo Fisher Scientific, Waltham, MA, USA).

### 4.4. Nanopore Sequencing

Monocytes isolated from buffycoats were lysed in TRIzol (Thermo Fisher) directly or after LPS activation. mRNA was isolated using the dynabeads mRNA Purification Kit (Thermo Fisher). A total of 1 µg mRNA per sample was loaded on MinION flowcells and run for approximately two hours. Raw data was interpreted by Nanopore Technologies sequencing base calling software Guppy version 3.2.10 (quality metrics in Supplementary Table S1), and quality control of the reads was performed using FastQC (version 0.11.8) [30]. Reads were aligned to the human genome (version GRCh38) using Minimap2 version 2.17 [15] with the recommended parameters for long-read RNA sequencing alignment (-ax splice -k14). The default FLAIR (version 1.4.0) pipeline [30] was used to generate a database of expressed isoforms in monocytes. First, FLAIR correct was executed with the –nvrna parameter, the reference ENSEMBL annotations (version 38.100) and additional junctional information (–shortread). The short-read junctional information was extracted from the STAR (version 2.7.1) [31] junction output of the next generation sequencing dataset with a custom script that keeps strand-specific information. Then, FLAIR collapse was executed with the non-default parameters –filter nosubset and –stringent. Finally, reads per isoforms were counted using flair quantify [30].

### 4.5. Next Generation Sequencing

Total RNA was isolated from stimulated cells with the NucleoSpin RNA II kit (Macherey-Nagel, Düren, Germany) following manufacturer instructions. RNA concentration was measured with a NanoDrop ND-1000 spectrophotometer (Peqlab Biotechnologie GmbH, Erlangen, Germany). For reverse transcription and cDNA synthesis with RevertAid H Minus transcriptase (Fermentas, Vilnius, Lithuania) 1 µg RNA was used. RNA libraries were prepared for 80 cycles single-read sequencing with the NEBNext Ultra RNA Library Prep Kit for Illumina^®^ following manufacturer instructions. Quality control was performed with Kapa quantification and a 2100 Bioanalyser (Kapa Biosystems and Agilent Technologies, Palo Alto, CA, USA). Sequencing was run on an Illumina^®^ NextSeq 500 system at the Utrecht Sequencing Facility, which produced single end reads of 75 bp (quality metrics in Supplementary Table S1). Quality control of the reads was performed using FastQC (version 0.11.8) [32]. Adaptors were trimmed using Trim Galore/Cutadapt (version 0.6.5) [33], and only reads with an average quality score (Q) above 20 and a minimum read length of 20 nucleotides were retained. Reads were aligned to the human genome (version GRCh38) and transcriptome (ENSEMBL version 38.100) with STAR (version 2.7.1) [31].

### 4.6. Transfection of HEK293T Cells with WTAP Isoforms

HEK293T cells (ATCC, CRL-3216; Cellosaurus: CVCL_0063) were obtained from the UMC Utrecht cell culture facility and maintained in DMEM (Thermo Fisher Scientific, Waltham, MA, USA) supplemented with 10% FBS (Thermo Fisher Scientific, Waltham, MA, USA) and 1% penicillin/streptomycin (Thermo Fisher Scientific, Waltham, MA, USA) under standard conditions. cells were seeded in 6-well plates (ScienCell Research Laboratories, Carlsbad, CA, USA; Cat# 0373) at a final volume of 3 mL DMEM per well and transfected at approximately 70% confluence using PEI MAX transfection reagent (Polysciences, Warrington, PA, USA). For each condition, 1.5 µg of plasmid DNA (pLV-WTAP1.1, pLV-WTAP1.4, or pLV empty vector) was diluted in 50 µL serum-free DMEM (Mix A), and 7.5 µL of PEI MAX was diluted in 50 µL DMEM (Mix B). Mix A and Mix B were combined, incubated for 10 min at room temperature, and added to the cells. Medium was refreshed 24 h after transfection. After 48 h, transfection efficiency was confirmed by GFP fluorescence. Cells were washed with phosphate-buffered saline (PBS) (Thermo Fisher Scientific, Waltham, MA, USA) and lysed in 100 µL Laemmli buffer. Lysates were boiled at 95 °C for 10 min and stored at −20 °C for Western blot analysis.

### 4.7. Statistical Analysis

All statistical analyses were performed using GraphPad Prism 10 for Windows, version 10.4.0 (621). All experiments were performed on CD14^+^ monocytes from at least three independent healthy donors (biological replicates, *n* = 3), unless otherwise indicated. qPCR assays were run in technical duplicates. Data are presented as mean ± standard error of the mean (SEM). Statistical comparisons were performed using paired two-tailed Student’s *t*-test for two-group comparisons, or one-way analysis of variance (ANOVA) with Tukey’s post hoc test for multiple groups. *p* < 0.05 was considered statistically significant.

## Data Availability

The original contributions presented in this study are included in the article/Supplementary Materials. Further inquiries can be directed to the corresponding author.

## Supplementary Materials

The following supporting information can be downloaded at: https://www.mdpi.com/article/10.3390/ijms26199364/s1.

## References

[1] Teng Y., Yi J., Chen J., Yang L. (2023). N6-Methyladenosine (m6A) Modification in Natural Immune Cell-Mediated Inflammatory Diseases. J. Innate Immun..

[2] Wen L., Sun W., Xia D., Wang Y., Li J., Yang S. (2022). The m6A methyltransferase METTL3 promotes LPS-induced microglia inflammation through TRAF6/NF-κB pathway. NeuroReport.

[3] Yu R., Li Q., Feng Z., Cai L., Xu Q. (2019). m6A reader YTHDF2 regulates LPS-induced inflammatory response. Int. J. Mol. Sci..

[4] Tong J., Wang X., Liu Y., Ren X., Wang A., Chen Z. (2021). Pooled CRISPR screening identifies m6A as a positive regulator of macrophage activation. Sci. Adv..

[5] Liu Y., Liu Z., Tang H., Shen Y., Gong Z., Xie N. (2019). The N6-methyladenosine (m6A)-forming enzyme METTL3 facilitates M1 macrophage polarization through the methylation of STAT1 mRNA. Am. J. Physiol. Cell Physiol..

[6] Wang J., Yan S., Lu H., Wang S., Xu D. (2019). METTL3 attenuates LPS-induced inflammatory response in macrophages via NF-κB signaling pathway. Mediat. Inflamm..

[7] Bai Y., Jiao X., Hu J., Xue W., Zhou Z., Wang W. (2023). WTAP promotes macrophage recruitment and increases VEGF secretion via N6-methyladenosine modification in corneal neovascularization. Biochim. Biophys. Acta Mol. Basis Dis..

[8] Li J., Wei L., Hu K., He Y., Gong G., Liu Q., Zhang Y., Zhou K., Guo J., Hua Y. (2024). Deciphering m^6^A methylation in monocyte-mediated cardiac fibrosis and monocyte-hitchhiked erythrocyte microvesicle biohybrid therapy. Theranostics.

[9] Li L.J., Fan Y.G., Leng R.X., Pan H.F., Ye D.Q. (2018). Potential link between m6A modification and systemic lupus erythematosus. Mol. Immunol..

[10] Luo Q., Fu B., Zhang L., Guo Y., Huang Z., Li J. (2020). Decreased Peripheral Blood ALKBH5 Correlates with Markers of Autoimmune Response in Systemic Lupus Erythematosus. Dis. Markers.

[11] Luo Q., Gao Y., Zhang L., Rao J., Guo Y., Huang Z., Li J. (2020). Decreased ALKBH5, FTO, and YTHDF2 in Peripheral Blood Are as Risk Factors for Rheumatoid Arthritis. BioMed Res. Int..

[12] Maini R.N., Elliott M.J., Brennan F.M., Feldmann M. (1995). Beneficial effects of tumour necrosis factor-alpha (TNF-α) blockade in rheumatoid arthritis (RA). Clin. Exp. Immunol..

[13] Mo X.B., Zhang Y.H., Lei S.F. (2018). Genome-wide identification of N6-methyladenosine (m6A) SNPs associated with rheumatoid arthritis. Front. Genet..

[14] Shi W., Zheng Y., Luo S., Li X., Zhang Y., Meng X., Huang C., Li J. (2021). METTL3 Promotes Activation and Inflammation of FLSs Through the NF-κB Signaling Pathway in Rheumatoid Arthritis. Front. Med..

[15] Li H. (2018). Minimap2: Pairwise alignment for nucleotide sequences. Bioinformatics.

[16] Ge Y., Chen R., Ling T., Liu B., Huang J., Cheng Y., Lin Y., Chen H., Xie X., Xia G. (2023). WTAP, transcriptionally regulated by p65, promotes inflammation through m^6^A modification and phase separation. Front. Immunol..

[17] Castro-Mondragon J.A., Riudavets-Puig R., Rauluseviciute I., Lemma R.B., Turchi L., Blanc-Mathieu R., Lucas J., Boddie P., Khan A., Pérez N.M. (2022). JASPAR 2022: The 9th release of the open-access database of transcription factor binding profiles. Nucleic Acids Res..

[18] Pierce J.W., Schoenleber R., Jesmok G., Best J., Moore S.A., Collins T., Gerritsen M.E. (1997). Novel Inhibitors of Cytokine-induced IκB Phosphorylation and Endothelial Cell Adhesion Molecule Expression Show Anti-inflammatory Effects in Vivo. J. Biol. Chem..

[19] Fan Y., Li X., Sun H., Gao Z., Zhu Z., Yuan K. (2022). Role of WTAP in Cancer: From Mechanisms to the Therapeutic Potential. Biomolecules.

[20] Han H., Fan G., Song S., Jiang Y., Qian C.A., Zhang W., Su Q., Xue X., Zhuang W., Li B. (2021). piRNA-30473 contributes to tumorigenesis and poor prognosis by regulating m6A RNA methylation in DLBCL. Blood.

[21] Weng L., Qiu K., Gao W., Shi C., Shu F. (2020). LncRNA PCGEM1 accelerates non-small cell lung cancer progression via sponging miR-433-3p to upregulate WTAP. BMC Pulm. Med..

[22] Zhou X., Chang Y., Zhu L., Shen C., Qian J., Chang R. (2021). LINC00839/miR-144-3p/WTAP (WT1 Associated protein) axis is involved in regulating hepatocellular carcinoma progression. Bioengineered.

[23] Ge J., Liu M., Zhang Y., Xie L., Shi Z., Wang G. (2022). SNHG10/miR-141-3p/WTAP axis promotes osteosarcoma proliferation and migration. J. Biochem. Mol. Toxicol..

[24] Li J.R., Liu L., Luo H., Chen Z.G., Wang J.H., Li N.F. (2021). Long Noncoding RNA DUXAP8 Promotes Pancreatic Carcinoma Cell Migration and Invasion Via Pathway by miR-448/WTAP/Fak Signaling Axis. Pancreas.

[25] Huang Q., Mo J., Liao Z., Chen X., Zhang B. (2022). The RNA m6A writer WTAP in diseases: Structure, roles, and mechanisms. Cell Death Dis..

[26] Deng J., Zhang J., Ye Y., Liu K., Zeng L., Huang J., Pan L., Li M., Bai R., Zhuang L. (2021). *N^6^*-methyladenosine–Mediated Upregulation of WTAPP1 Promotes WTAP Translation and Wnt Signaling to Facilitate Pancreatic Cancer Progression. Cancer Res..

[27] Ou B., Liu Y., Yang X., Xu X., Yan Y., Zhang J. (2021). C5aR1-positive neutrophils promote breast cancer glycolysis through WTAP-dependent m6A methylation of ENO1. Cell Death Dis..

[28] Cho S., Lee G., Pickering B.F., Jang C., Park J.H., He L., Mathur L., Kim S.S., Jung S., Tang H.W. (2021). mTORC1 promotes cell growth via m(6)A-dependent mRNA degradation. Mol. Cell.

[29] Xiao H., Zhang Y., Sun L., Zhao Z., Liu W., Luo B. (2021). EBV downregulates the m6A “writer” WTAP in EBV-associated gastric carcinoma. Virus Res..

[30] Tang A.D., Soulette C.M., van Baren M.J., Hart K., Hrabeta-Robinson E., Wu C.J., Brooks A.N. (2020). Full-length transcript characterization of SF3B1 mutation in chronic lymphocytic leukemia reveals downregulation of retained introns. Nat. Commun..

[31] Dobin A., Davis C.A., Schlesinger F., Drenkow J., Zaleski C., Jha S., Batut P., Chaisson M., Gingeras T.R. (2013). STAR: Ultrafast universal RNA-seq aligner. Bioinformatics.

[32] Wingett S.W., Andrews S. (2018). FastQ Screen: A tool for multi-genome mapping and quality control. F1000Research.

[33] Martin M. (2011). Cutadapt removes adapter sequences from high-throughput sequencing reads. EMBnet J..

