# Supplementary material for: Transcriptional Regulation of WTAP Isoforms by NF-κB Signaling in Human Monocytes
Source: Int J Mol Sci. 2025 Sep 25;26(19):9364. doi: 10.3390/ijms26199364 (PMC12524415; doi:10.3390/ijms26199364)
Supplement: Supplementary file 1 [file ijms-26-09364-s001.zip › ijms-3820239-supplementary.pdf]

Supplementary Table

Supplemental Table 1 - Quality metrics of RNA sequencing samples

Technique	Donnor	Condition	Total Reads	Average Read Depth per Gene
Shor-read RNA-Seq	HC1	Ex vivo	13051507	30.40058
Shor-read RNA-Seq	HC1	LPS	13423600	36.35849
Shor-read RNA-Seq	HC2	Ex vivo	11960933	25.58484
Shor-read RNA-Seq	HC2	LPS	12362491	33.25824
Shor-read RNA-Seq	HC3	Ex vivo	22877246	46.71664
Shor-read RNA-Seq	HC3	LPS	13572230	43.16934
Shor-read RNA-Seq	HC4	Ex vivo	11856167	27.95493
Shor-read RNA-Seq	HC4	LPS	14195670	39.87464
Shor-read RNA-Seq	HC5	Ex vivo	13159595	26.21978
Shor-read RNA-Seq	HC5	LPS	13002850	30.93351
Shor-read RNA-Seq	HC6	Ex vivo	11667742	26.3107
Shor-read RNA-Seq	HC6	LPS	15637546	39.22284
Shor-read RNA-Seq	HC7	Ex vivo	15591756	40.6352
Shor-read RNA-Seq	HC7	LPS	16746089	53.20491
Long-read RNA-Seq	HC10	Ex vivo	807248	13.21829
Long-read RNA-Seq	HC10	LPS	2474967	39.24452
Long-read RNA-Seq	HC8	Ex vivo	1304363	30.26601
Long-read RNA-Seq	HC8	LPS	1567068	35.08637
Long-read RNA-Seq	HC9	Ex vivo	1999571	51.79119
Long-read RNA-Seq	HC9	LPS	1768167	44.03258

Supplementary Table S1. Quality metrics of RNA sequencing samples. $CD14^{+}$ monocytes were isolated from healthy donors (HC) and cultured ex vivo or stimulated with 100 ng/mL LPS for 3 h**.** Libraries were prepared and sequenced on the Illumina NextSeq500 platform (single-end, 75 bp reads). Shown are the total reads obtained per sample and the average read depth per gene after quality filtering (Phred score > 20, read length > 20 bp).

Supplementary Figures

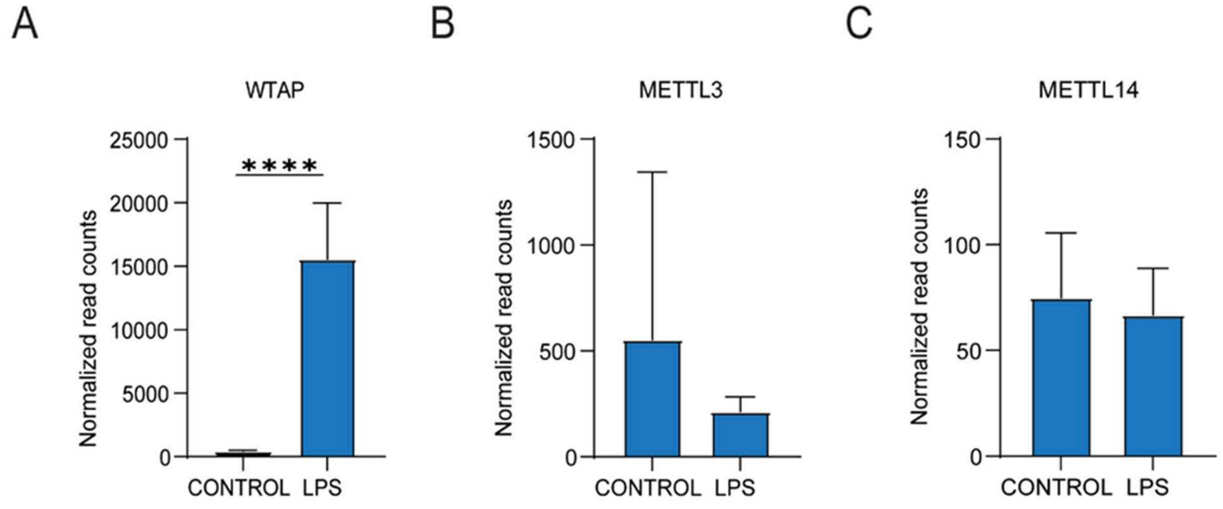

Supplementary Figure 1. Expression of WTAP, METTL3, and METTL14 in human monocytes. RNA-seq read counts in $CD14^+$ monocytes either ex vivo (control) or stimulated with LPS for 3 h**.** Data are presented as mean ± SEM (n = 6 per condition). (A) WTAP, (B) METTL3, (C) METTL14. Statistical analysis by paired Student's t-test; ****p < 0.0001.

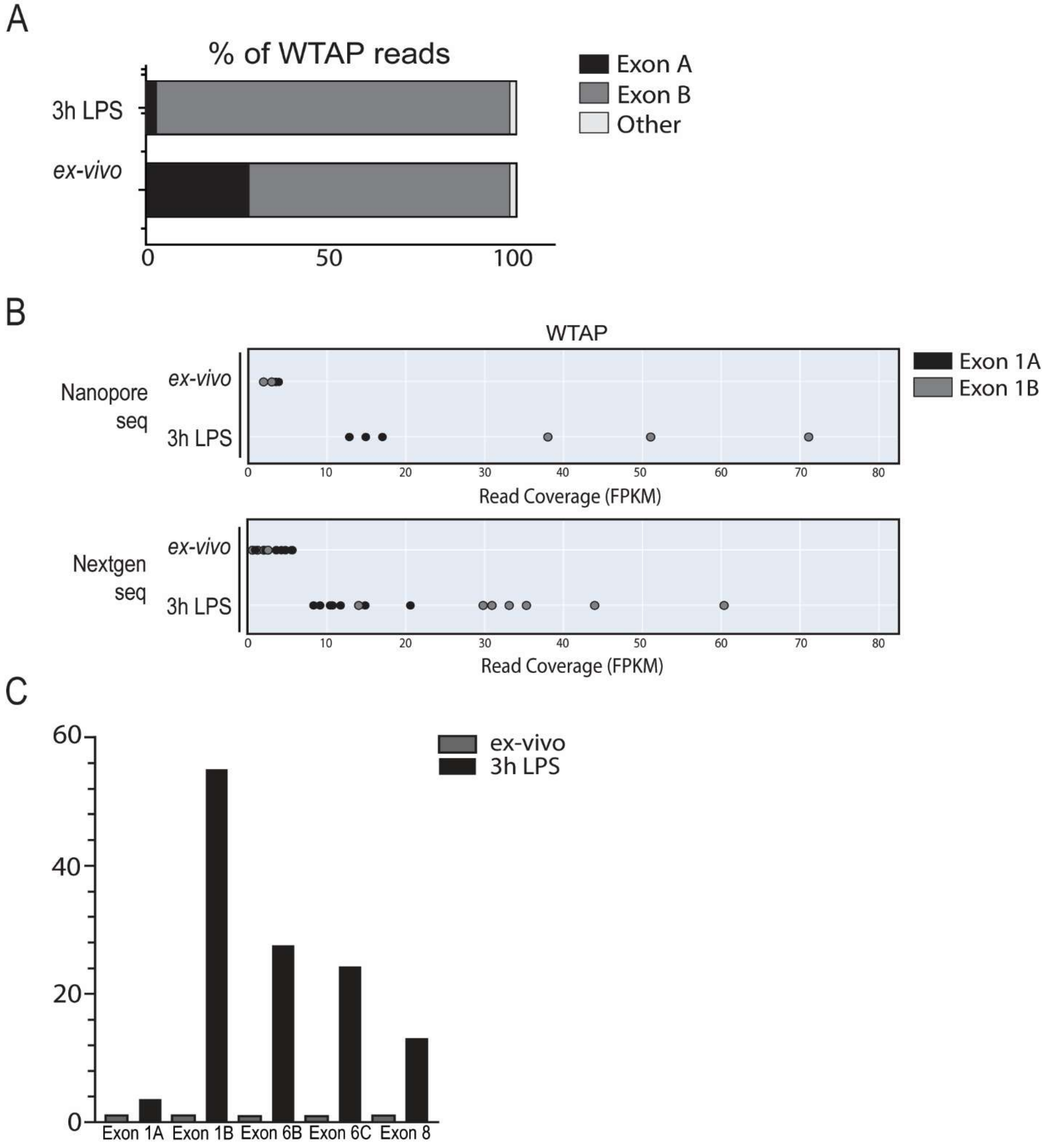

Supplementary Figure 2: Expression of specific WTAP mRNA isoforms is increased during monocyte activation. $CD14^{+}$ primary human monocytes were activated with 100 ng/mL LPS for 3 hours. (A) RNA-sequencing analysis demonstrating the percentage of WTAP exon 1A and exon 1B reads relative to total WTAP reads in ex vivo and activated monocytes. (B) Nanopore long-read and next-generation sequencing analysis showing read coverage for WTAP exon 1A and exon 1B in ex vivo and activated monocytes. (C) qPCR analysis of WTAP exon 1A, exon 1B, exon 6B, exon 6C, and exon 8 expression in ex vivo and activated monocytes.

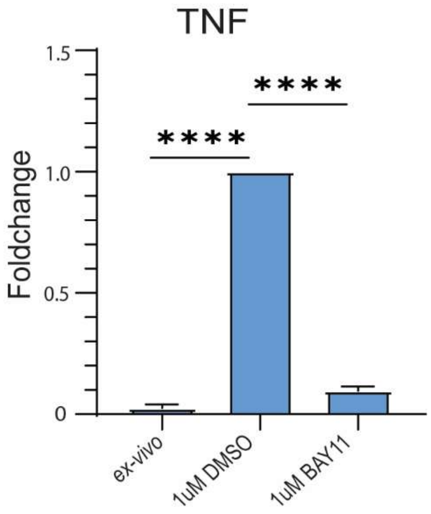

Supplementary Figure 3. LPS-induced TNF is reduced by the NF-κB inhibitor BAY 11-7082. CD14$^+$ monocytes from 3 independent healthy donors were directly lysed (ex-vivo) or stimulated with LPS (3 h) in the presence of BAY 11-7082 (1 µM or control (DMSO). TNF expression was quantified and normalized per donor to the DMSO control. Data are shown as mean ± SEM. Statistical significance was assessed using one-way ANOVA (*p < 0.05; **p < 0.01; ***p < 0.001; ****p < 0.0001).
